# Editorial

**Published:** 1844-08-24

**Authors:** 


### THE MEDICAL EXAMINER.

PHILADELPHIA, AUG. 24, 1844.

A subscriber in the West has kindly sent us the "Morning Courier" (a *political newspaper*, published at Louisville, Kentucky) of the 16th, 17th, and 18th ult., containing a long and scurrilous attack on us for some Editorial remarks on the subject of Medical teaching, in a former number of the Examiner. Although the writer had not honor and self-respect enough to restrain him from writing and publishing such a tirade of vulgar personalities, we are glad to find that he had sufficient to make him ashamed to put his name to it.

SYDENHAM SOCIETY.

We learn, that two of the valuable works published by the Sydenham Society for 1843, will soon be in the hands of their subscribers in this country; and that the third work is probably ready by this time. Dr. Dunglison has not yet received the annual report, but in a letter to him from Dr. Bennett, dated London, July 12, 1844, it is stated, that according to it the subscription for 1844 is now due. "We shall not, of course," says Dr. Bennett, "enforce the fine, &c. of this rule on you, on the other side of the water. At the same time, please remember, that unless the subscriptions for the second year are paid soon, there will be a chance of our not being able to supply the unpaid with books. You will at once see that unless we fix some time for payment, we cannot tell how many copies to print nor what funds we have to spend." "We ought not," he adds, "to complain of the slight difference in the subscription arising from the relation of the five dollar note to our £1 1*s.*; your 80 dollars will yield the society £15 9*s.* 3*d.* for the 16 subscriptions, after all expenses of remittance, &c. are discharged. This is quite as near as we can expect." It will be thus seen, that the subscribers on this side the Atlantic receive the books from the society in London at a less price than the English members.

The number of subscribers for 1843 has been so great, that the society are only able to furnish copies of the works published for that year to the *twelve* names first handed in by Dr. Dunglison. "You will see," says Dr. Bennett in his letter to that gentleman, "by the report, p. 23, how we were situated at the time of the last annual meeting in May, as regards the number of books on hand. The fact is, I have not, by a great many, sufficient to supply all who wish to join the society for the first year; and I am sorry to say, that I have not enough to supply all the persons whose names you have sent me. I have, however, sent you 12 copies of each of the works already issued, for the 12 names first in order in the lists sent in by you. The others we shall enter as subscribers for the current year, that is 1844-5. There will be a third volume for the 12 first year's subscribers, which, I hope will be ready to go by the end of the month. It is now in the binder's hands. A first volume for the current year will be out in about two months, in all probability." As soon as the report arrives we shall notice it; and a copy of it will doubtless be furnished by the Honorary Local Secretary to each of the subscribers.

It may be proper to add, for the information of the parties concerned, that the twelve subscribers referred to by Dr. Bennett sent in their names to Dr. Dunglison prior to the middle of June. Those who subscribed afterwards belong to the subscription year of 1844-5.

When the books arrive, due notice will be given to the subscribers.

It is somewhat strange to us, that there should be hesitation on the part of any one to subscribe to this important undertaking. In no other manner can the same amount of valuable matter be obtained so reasonably. Five dollars per annum procures from the society, at least three works that could not be obtained in any other manner; and hence it is not astonishing, that the influx of British members for 1843 has been so great as to render it impracticable for the society to supply them all.

### DISEASES OF THE MISSISSIPPI VALLEY.

From various sources, but more especially from a note by Professor Drake, published in the *New Orleans Medical Journal,* we are informed that that distinguished gentleman is engaged in collecting materials for "a history of the prevalent maladies of the Mississippi Valley." We regard this as a highly important announcement to the profession. Nothing could be more opportune than such a work from such a source. No man is more capable of preparing a faithful account of the diseases of that section of the country than Dr. Drake, and this we doubt not is the opinion of the profession.—For ourselves, we shall look for it with particular interest. We have so many contradictory accounts from essayists,—sometimes perhaps from want of knowledge to appreciate what they see, but more generally, it is probable, because of the modifications which occur in different years and seasons of the year,—that we are at a loss as to the degree of credit that should be attached to any thing we meet with on the subject.

When the book of Prof. Drake appears, we shall have a standard—something that will enable us to appreciate the extravagant and nonsensical prating about the giant diseases of the West and South of which we have heard so much—often from men who, very unlike Professor Drake, possess neither the capacity, the habits, nor the opportunities for observation, which are necessary to command the confidence of the profession. In short, we shall be able to judge whether diseases and their treatment, which are every where else amenable to the well established laws of pathology and therapeutics, are in the Valley of the Mississippi an exception.

---

### THE BOSTON MEDICAL AND SURGICAL JOURNAL.

On the 7th of the present month, this popular periodical entered upon its *thirty-first* volume. In announcing the fact, the Editor, in appropriate and feeling language, adverts to the toil and solicitude which the Journal has cost him in the years that have past, and gratefully acknowledges the "untiring kindness, assistance and patronage" by which he has been encouraged and sustained.

"Journals of medicine, (he remarks) like other enterprises, have greatly increased, as the natural result of the increased facilities and resources of a great and prosperous nation. Some, indeed, have only breathed to die; and others, after having maintained for a while a sickly existence, were compelled to yield to the force of circumstances, and finally disappear. Ours is now the only one in the United States which is published weekly, having survived, unharmed, the rivalry of no less than three publications of the same class. It requires something more than a prospectus of operations, to maintain a medical journal. There is necessarily a fearful outlay of capital, quite discouraging at first; and when there is taken into account the great number of losses annually occurring, very few, it is presumed, would be willing to enter anew upon the business, after having had experience in permanently establishing one. Unlike other periodicals, its subscribers are of necessity only here and there one out of hundreds and thousands, and then they are spread so widely over the entire face of the Union, that collections are always difficult. Still, under all the aspects of the case, we have passed on till the commencement of this *thirty-first volume.* We hope for the continued good will, and the literary and scientific assistance of our brethren. With their continuance, and our own continued exertions, the Journal will pursue its quiet way, without ostentation, or a presumptuous display unbecoming the legitimate object to which it is expressly devoted, or the character it has attained."

Our estimable brother need have no apprehensions for the future. His enterprise is no longer an experiment. The industry, talents, and imperturbable good nature which he has heretofore displayed, are a guaranty which will secure for him the continued support of an enlightened and generous profession.

---

### WESTERN JOURNAL OF MEDICINE AND SURGERY.

Just as our present number was going to press, and after the greater part of the matter was in type, we received the August number of the respectable Journal above named, in which we find an article, referring to the disreputable publication alluded to in another column, that calls for a reply. Under the caption of "*Medical Examiner and Western Medical Schools,*" the following remarks occur, written by one of the editors:—

"We must say that we consider the allusion to *Western Medical Schools* by the editor of the Medical Examiner, in the number of that paper for June 29th, an unfortunate one. We hope without intending it, he has made a charge against his brethren in this region which must give general and deep offence."

We are no admirer of obstinacy, nor of those who affect infallibility; nothing indeed affords us more pleasure, when properly called upon, than to explain or retract any thing that we may have said or done, calculated to give unprovoked offence. The remarks, above referred to, are not *discourteous* and they are not *anonymous,* and we have therefore no hesitation in making the proper reply. We say, then, with perfect frankness, that since the Editor thinks our remarks of the 29th of June are calculated to produce the impression, that we meant to charge either dishonor or incompetency on the *Western Medical Schools,* our remarks *were* "unfortunate." We had no such design. The Editors of the *New Orleans Medical Journal* asserted, in substance, the doctrine which we have understood to be perseveringly promulgated by *one* individual in the West for years past, that those who study their profession "in the capitals of Europe and the United States," are unprepared to practise medicine in the Valley of the Mississippi, until they have established for themselves "*a new code of principles and practice.*" This doctrine we regarded, and still do regard, as degrading to the science. The New Orleans Journal furthermore asserts that "the Medical Profession has been for sometime gradually losing caste and respectability in the South—that unworthy and incompetent members are constantly gaining admission into the ranks—and that the charlatan and empiric annually find it less difficult to maintain a successful competition with the licensed practitioner." We mentioned the fact, that since the establishment of Medical Schools in the Valley of the Mississippi, comparatively few young men from that region seek instruction elsewhere, under the belief that there only can they learn the peculiar diseases of the South; and, consequently, we inferred that if the profession in that quarter was "gradually losing caste," the Medical "Schools in the Capitals of Europe and the United States," were certainly guiltless of contributing to the unfortunate result.—Our object was to protest against what we regarded as false teaching, calculated to contract the views of ingenuous youth; but we had no intention whatever of casting any imputations of a dishonorable character upon the Western or any other *Schools.* In nearly all of them we have personal friends—men whom we know to be honorable, liberal-minded, and intelligent—and we know how to separate such from individuals of a different stamp. In conclusion, we have only to add our extreme regret that the able and respectable Editors of the *Western Journal of Medicine and Surgery* should have thought it necessary for them, *under any circumstances,* to admit to their pages language in relation to us which one of them confesses he "should avoid,"—such as *he* certainly never employs;—and that language, too, extracted from an anonymous contribution to a newspaper!

PHILADELPHIA DISPENSARY.

At a meeting of the Managers of this Institution on the 20th inst., Charles Huston, M. D., was elected one of the Physicians, in the place of William B. Page, M. D., resigned.

We have received the Annual Announcement of the Willoughby University of Lake Erie, for the course of lectures in the Medical Department for 1844-5.

The Professors are:

Amasa Trowbridge, M. D., Professor of Surgery.

George McCook, M. D., Adjunct Professor of Surgery.

Henry H. Childs, M. D., Professor of Obstetrics, &c.

James Quackenboss, M. D., Professor of Anatomy and Physiology.

Robert H. Paddock, M. D., Professor of Chemistry and Pharmacy, and Materia Medica.

John Butterfield, Professor of Theory and Practice, &c.

Isaac J. Allen M. D., Counsellor at Law, Professor of Medical Jurisprudence.

Professors Childs and Trowbridge are veteran practitioners, as well as teachers, whose reputation must prove advantageous to the school. "The College is located in the village of Willoughby, Lake County, nineteen miles east of Cleaveland, and fourteen west of Fairport."

